# Foxp3^+^ Regulatory T Cells Control Persistence of Viral CNS Infection

**DOI:** 10.1371/journal.pone.0033989

**Published:** 2012-03-20

**Authors:** Dajana Reuter, Tim Sparwasser, Thomas Hünig, Jürgen Schneider-Schaulies

**Affiliations:** 1 Institute for Virology and Immunobiology, University of Würzburg, Würzburg, Germany; 2 Institute of Infection Immunology, TWINCORE, Centre for Experimental and Clinical Infection Research, Hannover, Germany; University of Texas Medical Branch, United States of America

## Abstract

We earlier established a model of a persistent viral CNS infection using two week old immunologically normal (genetically unmodified) mice and recombinant measles virus (MV). Using this model infection we investigated the role of regulatory T cells (Tregs) as regulators of the immune response in the brain, and assessed whether the persistent CNS infection can be modulated by manipulation of Tregs in the periphery. CD4^+^ CD25^+^ Foxp3^+^ Tregs were expanded or depleted during the persistent phase of the CNS infection, and the consequences for the virus-specific immune response and the extent of persistent infection were analyzed. Virus-specific CD8^+^ T cells predominantly recognising the H-2D^b^-presented viral hemagglutinin epitope MV-H_22–30_ (RIVINREHL) were quantified in the brain by pentamer staining. Expansion of Tregs after intraperitoneal (i.p.) application of the superagonistic anti-CD28 antibody D665 inducing transient immunosuppression caused increased virus replication and spread in the CNS. In contrast, depletion of Tregs using diphtheria toxin (DT) in DEREG (depletion of regulatory T cells)-mice induced an increase of virus-specific CD8^+^ effector T cells in the brain and caused a reduction of the persistent infection. These data indicate that manipulation of Tregs in the periphery can be utilized to regulate virus persistence in the CNS.

## Introduction

The role of CD4^+^ CD25^+^ regulatory T cells (Tregs) in autoimmune and pathogen-induced immune responses has been studied intensively during recent years. An important tool was provided by the discovery of the transcription factor Foxp3 (forkhead box P3) as a marker for Tregs and their suppressive activity [1], [2], [3], [4]. During acute viral infections depletion of Tregs was found to prevent the development of exhausted T cells and to improve the immune response. In addition, transient depletion of Tregs in several persistent viral infections led to reactivation of virus-specific T cells and reduction of the virus load [5], [6], [7], [8], [9]. The important protective role of Tregs against an overshooting immune response in the CNS became obvious in animal models of stroke and experimental autoimmune encephalitis [10], [11], and human immunodeficiency virus-1 (HIV-1)-associated neuro-degeneration, where they reduce astrogliosis and microglia-mediated inflammation [12]. Interestingly, some viruses even developed the strategy to support the expansion of Tregs in order to suppress anti-viral cytotoxic T cell (CTL) responses and to limit viral immunopathogenesis [13], [14], [15], [16], [17], [18]. Defects in regulation of numbers or the activity of Tregs are also involved in a number of human autoimmune diseases such as type 1 diabetes, rheumatoid arthritis, and multiple sclerosis [19].

Viral infections of the brain mostly represent clinically important, often life-threatening complications of systemic viral infections. For example, after acute measles, CNS complications may occur early as acute post-infectious encephalitis, or after years of viral persistence as subacute sclerosing panencephalitis (SSPE). Epidemiological studies indicated that the primary MV infection of SSPE patients takes place predominantly below the age of two years, when the immune system of the host is still immature and residual maternal antibodies may be absent or not sufficient for complete virus neutralization [20], [21]. For intracerebral MV infection of mice, a transgenic human receptor for MV is not necessarily required, and various infection models exist depending on the age of the mice, the virus strain, and the infectious dose [22], [23], [24], [25], [26]. As found in genetically unmodified and MV-receptor transgenic mice, T cells and IFN-γ have a critical role for protection and clearance of virus from the brain [27], [28], [29], [30]. Transient immunosuppression during MV-infection enhanced virus replication and facilitated persistence [31]. After intranasal infection of MV-receptor CD150-transgenic mice, a specific antiviral cellular immune response including an increased proportion of Foxp3^+^ Tregs in the spleen was observed [32]. Although the presence and activity of Tregs has been demonstrated, their actual role in viral immunosuppression or immunopathogenesis in the brain remains to be elucidated.

Here we investigated the role of Tregs for virus persistence in the CNS. According to our model, two week old C57BL/6 mice (an age in which these mice survive infection, while the immune system is still not fully matured) were intracerebrally infected and virus persists in a limited number of neurons in most animals for more than 10 weeks [33]. We expanded and depleted Tregs in the periphery during the persistent phase of the viral infection, and investigated whether this can be exploited to modulate the “hidden” CNS infection. Our data indicate that this is indeed the case and that manipulation of Tregs can be utilized to regulate virus-specific CD8^+^ effector T cells and virus persistence in the brain.

## Results

### Regulatory T cells are present predominantly in spleen and lymph nodes, but at a low frequency also in the brain

Two week old mice were intracerebrally (i.c.) infected with 10^3^ PFU recombinant MV expressing the rodent brain-adapted haemagglutinin CAMH and eGFP, or, when indicated, the recombinant MV without eGFP. Both recombinant viruses (rMV^Edtag^EGFP-CAMH and rMV^Edtag^CAMH) have the same distinct tropism for mouse neurons, and infections cause similar acute and persistent CNS infections [24], [33], [34], [35]. These recombinant viruses were designated throughout the manuscript as rMV-green and rMV.

**Figure 1:**
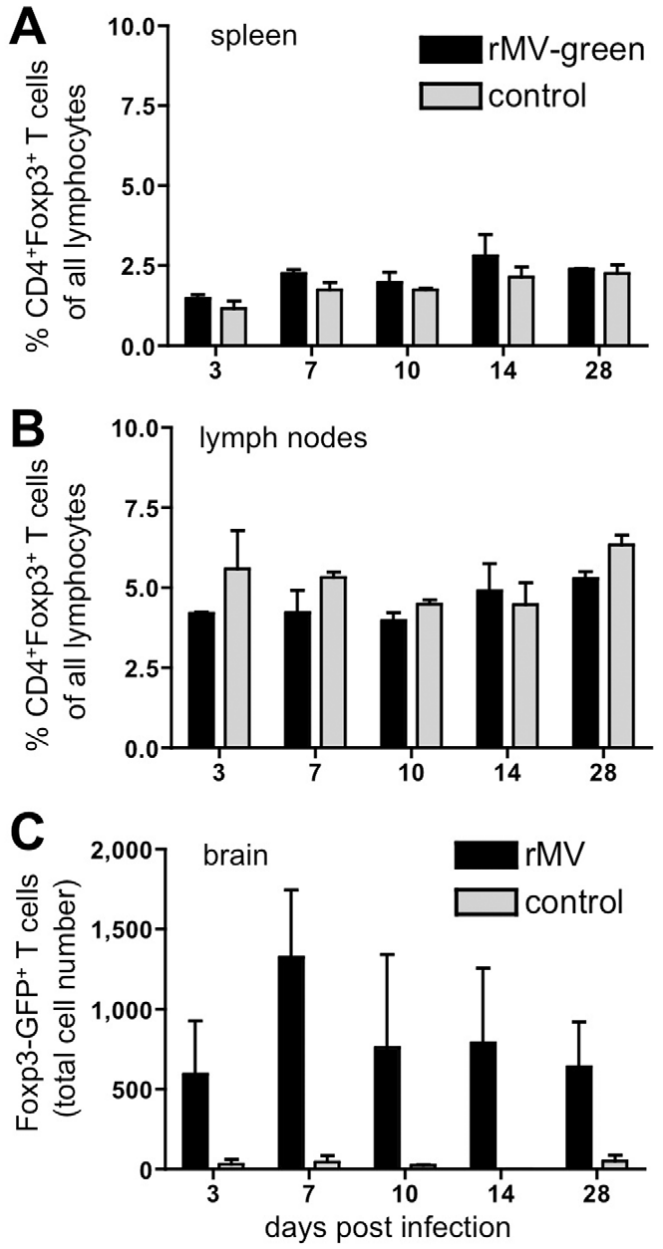
Detection of regulatory T cells in secondary lymphoid organs and the brain of C57BL/6 and DEREG mice. Mice were i.c. infected and analyzed at 3, 7, 10, 14, and 28 dpi as indicated. The percentages of regulatory CD4^+^ Foxp3^+^ T cells all lymphocytes in spleen (**A**) and draining cervical lymph nodes (CLN) (**B**) of rMV-green-infected and PBS-injected (ctrl) animals were determined using C57BL/6 mice. Foxp3^+^ T cells were quantified by flow cytometry after staining with antibodies to CD25, CD4, and Foxp3, and gating on positive cells. In brains (**C**), total cell numbers of Foxp3-GFP^+^ Tregs were determined using of rMV-infected and PBS-injected (ctrl) DEREG mice. Mean values ± SEM are presented (n = 3).

To determine the number of Tregs present in our infection model in secondary lymphoid organs and the brain, C57BL/6 mice were infected with rMV-green and analyzed 3, 7, 10, 14 and 28 days post infection. Lymphocytes were isolated from 6 draining cervical lymph nodes (LN), the spleen, and the brain of MV-infected and control (i.c. injected PBS) animals. The fraction of CD4^+^ CD25^+^ Foxp3^+^ T cells of all lymphocytes was determined to be 1.5–3% in the spleen and 4–6% in LN (Fig. 1 A, B) with no significant difference between infected and control animals. This corresponds to a total number of Tregs of approximately 1×10^6^ in the spleen and 2×10^5^ in the prepared LN at day 28 post infection. Only a small statistically not significant number of Foxp3^+^ T cells was detected in the brain, irrespective of the infection (data not shown). To obtain statistically significant results and to reduce standard errors caused by the staining procedure, we used DEREG mice, which express GFP in Foxp3^+^ cells. After infection of these mice with rMV (not expressing GFP), a small but significant number of GFP^+^ cells, approximately 800 per brain, was detected in the brains, whereas almost no GFP^+^ cells were detected in controls (Fig. 1 C; n = 3, P<0.05, two way ANOVA).

### Persistent CNS infection is controlled by the immune system

**Figure 2:**
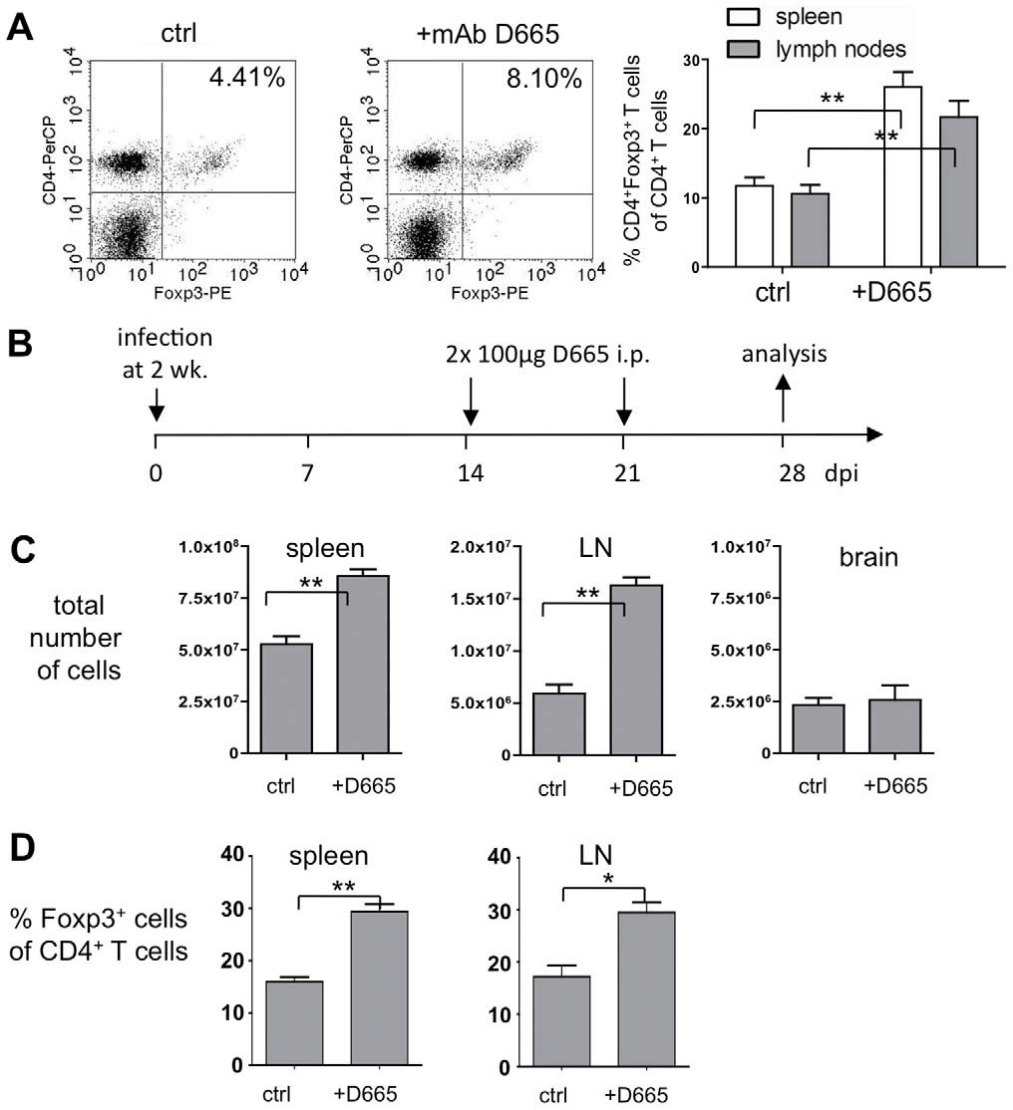
Expansion of T lymphocytes with the superagonistic CD28 antibody D665. (**A**) Two week old uninfected C57BL/6 mice were i.p. injected with 100 µg mAb D665 or PBS (control) and analyzed 3 days later. Lymphocytes were isolated from the spleen and lymph nodes (12 per mouse; 6 cervical, 4 axillary and 2 inguinal lymph nodes). FACS dot plot examples for CD4^+^ Foxp3^+^ T cells in the lymph nodes are shown (left panels: ctrl and +mAb D665 with percentages of all gated lymphocytes). Right panel: quantitative evaluation of the proportion of Foxp3^+^ T cells (percent of all CD4^+^ T cells) in spleen and lymph nodes of D665-treated and control animals (mean values ± SEM, n = 4, P<0.01). (**B**) Experimental setup used for the treatment of MV-infected mice with mAb D665. As a control an appropriate volume of PBS was injected. (**C**) The total number of lymphocytes in the spleen and draining lymph nodes (LN) (n = 3; P<0.01), and total number of percoll-isolated cells in brains of D665-treated and control animals (n = 3). (**D**) Quantitative evaluation of CD4^+^ Foxp3^+^ Tregs in the spleen and LN of D665-treated and control animals (percent CD4^+^Foxp3^+^ cells of all CD4^+^ T cells; n = 3, P<0.002 and P<0.02, respectively).

In order to find out whether Tregs have an influence on the persistent CNS infection we first expanded and functionally activated Tregs using the superagonistic anti-CD28 monoclonal antibody clone D665 (mAb D665) [36]. This mAb, while causing a transient expansion of the total lymph node and splenic T cell population, predominantly expands functional CD4^+^ Tregs without causing systemic cytokine release [37]. In order to test the efficiency of mAb D665 in young mice, we measured Tregs in a preliminary experiment in uninfected mice 3 days after a single i.p. injection of 100 µg D665 by flow cytometry. The proportion of CD4^+^Foxp3^+^ Tregs increased in the spleen and lymph nodes approximately 2-fold (Fig. 2 A; n = 4; P<0.01). This result indicated that mAb D665 can be used for expansion of Tregs also in young mice.

**Figure 3:**
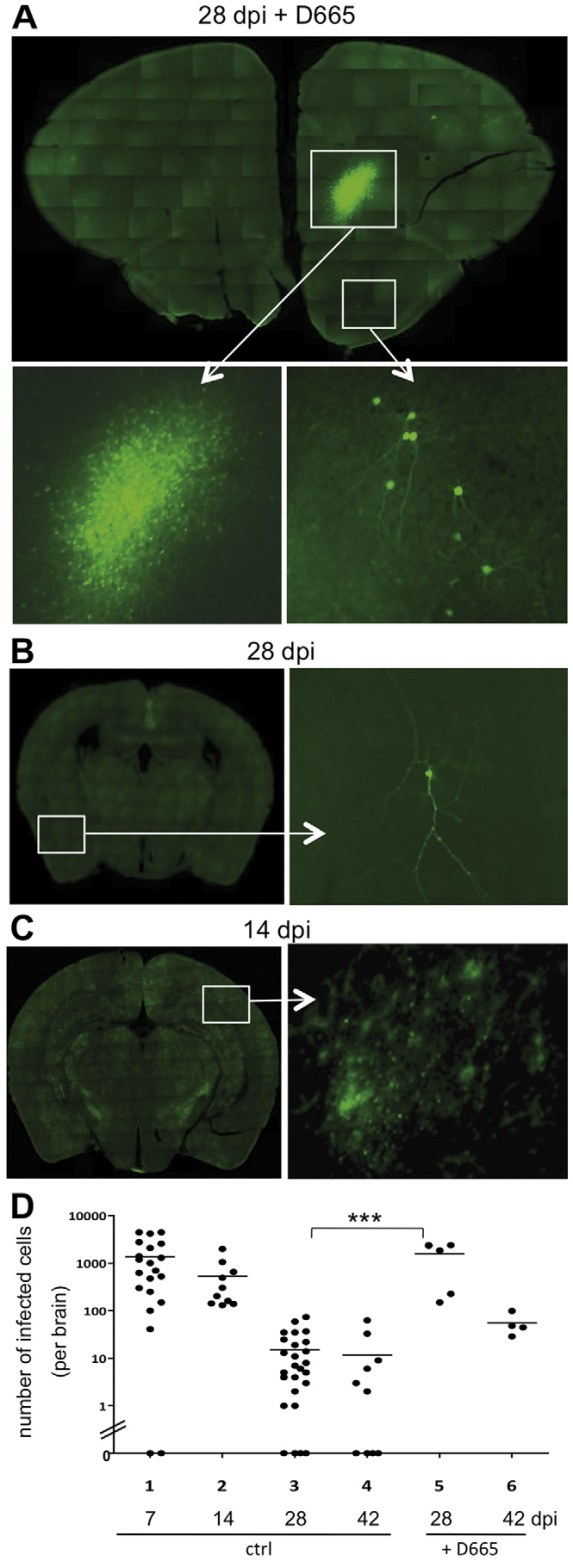
Expansion of T lymphocytes with the superagonistic CD28 antibody D665 induces virus replication and spread. Consecutive coronal brain sections (100 µm sections) were prepared from complete rMV-green-infected mouse cerebra and analyzed using the UV microscope. Overviews and details of a typical section of an infected brain of a mouse treated with mAb D665 and analyzed at 28 dpi (**A**), and sections of infected control animals in the absence of mAb D665 at 28 dpi (**B**), and 14 dpi (**C**) are shown. The numbers of infected eGFP^+^ cells per brain (sections through the complete cerebrum of each animal were evaluated as described [33]) were determined microscopically in infected control C57BL/6 mice at 7, 14, 28 and 42 dpi (**D**, lanes 1–4) and in D665-treated mice at 28 and 42 dpi (**D**, lanes 5 and 6). The difference between control and D665-treated mice at 28 dpi was highly significant (P<0,0001).

For the following experiments, to investigate the effect of mAb D665 on the persistent viral infection, we applied 100 µg at day 14 and 21 post infection, and analyzed the brains at 28 dpi (Fig. 2 B). Under these conditions the total number of lymphocytes increased significantly after D665 treatment in spleen and LN, but not in the brain (Fig. 2 C). Furthermore, the proportion of Foxp3^+^ Tregs increased significantly (approximately 2-fold, P<0.01) in spleen in LN (Fig. 2 D). Interestingly, histological analyzes revealed that the extent of the viral infection increased considerably after D665 treatment. Large clusters and groups of bright GFP-positive (directly reflecting virus replication) infected neurons emerged in the brains of these mice (Fig. 3 A). This was in striking contrast to the brains of untreated mice at 28 dpi, which contain only a limited number of infected neurons (Fig. 3 B). It is also strikingly different from brains at 14 dpi, when the treatment with D665 begins. At this time point areas with weak (vanishing) GFP expression are observed, that represent areas from which virus is being eliminated (Fig. 3 C). The quantification demonstrated approximately 100-fold more infected cells per brain in D665-treated animals in comparison to control animals at 28 dpi (Fig. 3 D, compare lanes 3 and 5; differences were highly significant: P<0.0001). Two weeks later, at 42 dpi, the number of infected cells was reduced, but still higher than in control animals (Fig. 3 D, compare lanes 4 and 6). The data indicate that CD28-mediated expansion of Tregs in the periphery during the persistent phase of the viral infection induced a transient release of the viral infection from immunological control resulting in a dramatic increase of virus replication and spread in the CNS.

### Transient depletion of Tregs stimulates infiltration of virus-specific T cells into the brain and virus elimination

**Figure 4:**
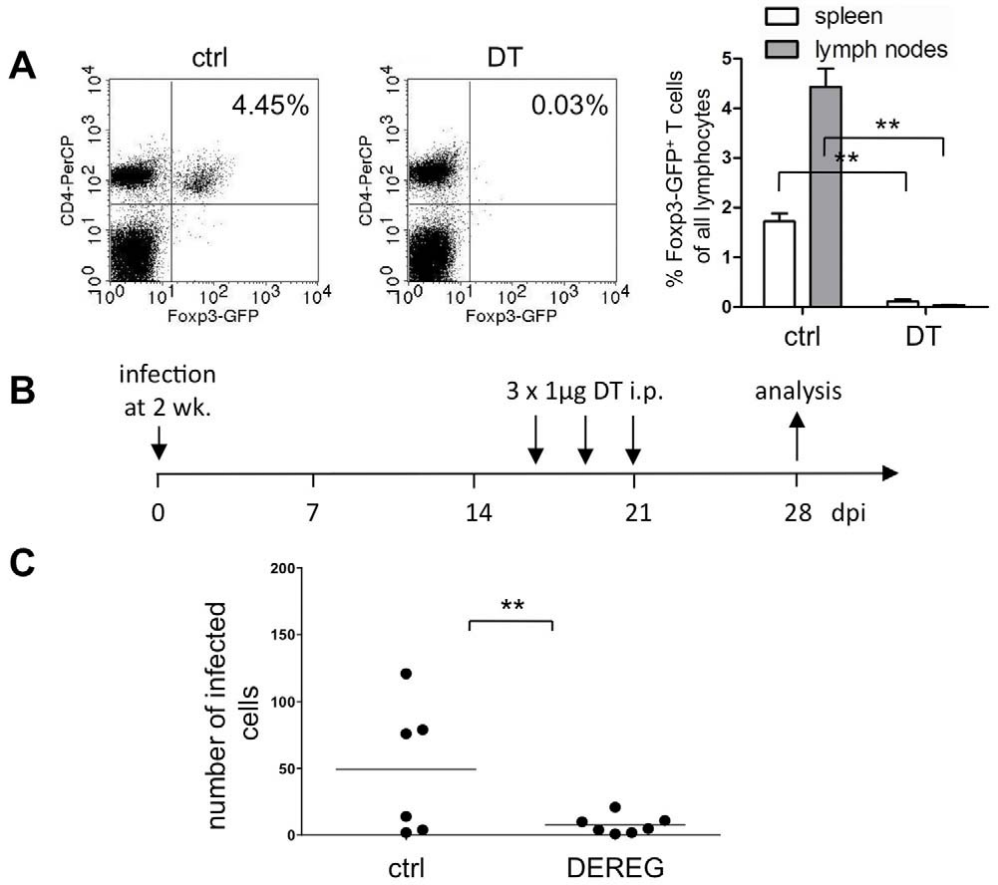
Depletion of Tregs leads to a reduction of the CNS infection. (**A**) Adult DEREG (DEREG^−/+^) mice were i.p. injected with 1 µg diphtheria toxin (DT) or with an appropriate volume of PBS (ctrl) at 6 consecutive days and analyzed the next day. Lymphocytes were isolated from the spleen and LN (6 cervical, 4 axillary, and 2 inguinal). FACS dot plot examples for regulatory CD4^+^ Foxp3-GFP^+^ T cells in the lymph nodes (left panels), and a quantitative evaluation of Foxp3-GFP^+^ T cells (percentage of all lymphocytes, right panel) from spleen and LN (mean values ± SEM, n = 4, P<0.01) are shown. (**B**) Experimental setup for the treatment of young MV-infected DEREG mice with DT at day 17, 18, and 20 post infection and analysis at 28 dpi. (**C**) Quantitative evaluation of the number of infected eGFP^+^ cells at 28 dpi in DEREG (DEREG^−/+^) and control (DEREG^−/−^) mice both infected i.c. with rMV-green and treated with DT. The reduction of mean values from 50 to 8 was significant, with P = 0,0098. The number of infected eGFP^+^ cells per brain was determined by microscopic evaluation of 100 µm sections through the complete cerebrum as described [33].

For the depletion of Tregs we used transgenic mice expressing the human diphtheria toxin (DT) receptor under the control of the Foxp3-promoter, which can be treated with DT to eliminate specifically Foxp3^+^ Tregs (DEREG-mice) [38]. To test the activity of the DT-batch used, adult DEREG mice were in a pilot experiment treated with DT under standard conditions (1 µg DT i.p. injected at 6 consecutive days) and analyzed the next day. Lymphocytes isolated from the spleen and lymph nodes were analyzed by flow cytometry to demonstrate the successful depletion of Foxp3^+^GFP^+^ Tregs by more than 90% (Fig. 4 A). In order to assess the effect of Treg depletion on virus persistence in young mice, persistently infected mice were treated at 3 consecutive days (at day 17, 18, and 20 post infection) with 1 µg DT and analyzed at day 28 post infection (Fig. 4 B). In these mice, the viral infection was again quantified histologically in subsequent brain slices through the complete cerebrum as described earlier [33]. Treg depletion in DEREG^−/+^ mice (compared to DT-treated DEREG^−/−^ mice as control animals) led to a significant reduction of the number of infected neurons from brains of persistently infected animals (n = 6, P = 0.0098; Fig. 4 C).

**Figure 5:**
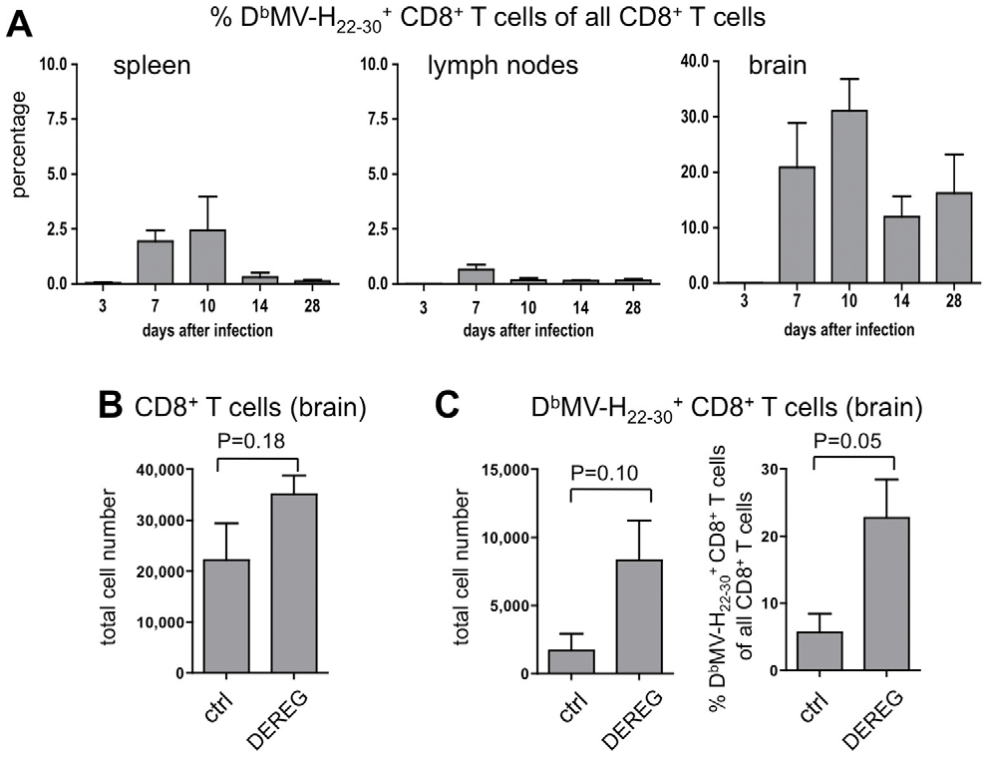
Treg depletion causes an increase in virus-specific CD8+ T cells in the brain. (**A**) The proportion of virus-specific D^b^MV-H_22–30_-pentamer^+^ CD8^+^ T cells of all CD8^+^ T cells was determined in spleen, lymph nodes, and brains of MV-infected C57BL/6 mice at days 3, 7, 10, 14, and 28 post infection (n = 3). MV-specific cells were gated as CD19-negative lymphocytes to exclude pentamer^+^ CD19^+^ cells. The total number of CD8^+^ T cells (**B**) and the number and proportion of D^b^MV-H_22–30_-pentamer^+^ CD8^+^ T cells (**C**) was determined in brains of 28 days infected control (DEREG^−/−^) and DEREG (DEREG^−/+^) mice, both treated with DT (Values ± SEM; n = 3).

In order to investigate whether this increase in virus clearance correlates with the presence of higher numbers of virus-specific CD8^+^ T cells, we first identified T cell receptor recognized peptides presented by MHC class I of C57BL/6 mice (H-2^b^). Two recognized peptides, D^b^MV-H_22–30_ (RIVINREHL) and D^b^MV-H_446–454_ (SNHNNVYWL), were identified as effective in stimulating IFN-γ synthesis using the ELISPOT assay (data not shown). MV-specific CD8^+^ T cells were then identified by flow cytometry using MHC class I (H-2D^b^) pentamers loaded with the most effective peptide MV-H_22–30_. The percentages of D^b^MV-H_22–30_-specific CD8^+^ T cells in spleen, LN, and the brain were determined after 3, 7, 10, 14 and 28 days post infection. Considerable proportions of D^b^MV-H_22–30_-pentamer-positive CD8^+^ T cells were detected in the brain at 7, 10, 14, and 28 dpi (Fig. 5 A). Interestingly, high percentages of D^b^MV-H_22–30_-specific CD8^+^ T cells are present in the brain during the persistent phase of the infection (at 28 dpi approximately 18% of all CD8^+^ T lymphocytes). Mock-treated animals (i.c. PBS injection; ctrl) did not contain virus-specific CD8^+^ T cells. After treatment of DEREG mice with DT, the number of Tregs decreased by approximately 95%, while the number of CD8^+^ T cells in brains slightly increased (Fig. 5 B). Interestingly, after depletion of Tregs, the fraction and absolute number of D^b^MV-H_22–30_-specific CD8^+^ T cells increased from 2,000 to 8,000 cells per brain, or 5% to 23% of CD8^+^ T cells (n = 3, P = 0,05; Fig. 5 C). Thus, depletion of Tregs during the persistent phase of infection led to an increase of virus-specific CD8^+^ T cells and a significant reduction of the persistent infection in the brain.

## Discussion

Our results indicate that the immune system keeps the “hidden” persistent viral CNS infection under permanent control. Manipulation of Tregs in the periphery had significant consequences for the fate of the viral infection in the brain, although only few infected neurons are present during the persistent phase of the infection. Expanding the number of Tregs by superagonistic CD28 antibody D665 led to an activation of virus replication and dramatic increase of the number of infected neurons, whereas transient depletion of Tregs by DT led to a significant reduction of the number of infected neurons. Interestingly, complete elimination of virus and clearance of the infection was not achieved by transient depletion of Tregs suggesting that additional means of an antiviral immune response are required for complete clearance.

Looking for Tregs in the brain, we did not detect a significant number of CD4^+^ Foxp3^+^ Tregs by FACS-analysis after staining with Foxp3-specific antibodies, and only a small number of GFP-expressing Foxp3^+^ cells in infected DEREG mice. These findings suggest that only very few Tregs, if any at all, are required within the brain for the regulation of effector immune cells, and that this regulation predominantly takes place in secondary lymphoid organs. Sellin et al. observed an increase of Foxp3^+^ Tregs as a consequence of MV infection of CD150-transgenic mice in spleen and brain [32], which suggests that also the MV-infection itself supports the expansion and migration of Tregs, and that these infection-induced Tregs may be part of the multifactorial MV-induced immunosuppression [3], [4]. Tregs can restrain effector T cell responses through the production of immunomodulatory cytokines, such as TGF-β, IL-10, and IL-35, expression of inhibitory ligands, such as CTLA-4 and LAG-3, cytokine consumption, and direct cytolysis. It remains to be elucidated which of these mechanisms are involved in the persistent brain infection with MV.

Several reports support the view that T cells play a decisive role in control of viral CNS infections. Using primary human lymphocytes, *in vitro* experiments demonstrated that CD8^+^ T lymphocytes control the dissemination of MV [39]. In resistant mouse strains the depletion of the CD4^+^ T cell subset by monoclonal antibodies led to a breakdown of resistance to the infection, whereas depletion of CD8^+^ T cells had no effect [23], [40]. In TAP-transporter deficient mice, which cannot present antigen on MHC class I molecules, MV was found to spread more transneuronally than in brains of normal mice [41]. These findings indicated that infected neurons are target cells of CD8^+^ lymphocytes, and that brain infections to some extent can be inhibited by CTL activity. Further investigations revealed that CD4^+^ T cells are able to protect either alone (in resistant mouse strains), or through cooperation with CD8^+^ T cells (in mice with intermediate susceptibility), and that CD8^+^ T cells are able to protect alone after immunization of the mice [42], [43]. Using CD46-transgenic Rag-deficient mice, adoptive transfer of lymphocytes revealed that the combined activity of CD4^+^ T lymphocytes with CD8^+^ T cells or B cells is required to control the intracerebral infection [44]. Thus, most findings support the view that CD8^+^ T cells play an important role in the control of transneuronal virus spread, and our findings suggest that MV-specific CD8^+^ T cells are involved in maintaining the steady state and control of infection during the persistent phase of CNS infection.

## Materials and Methods

### Animal infection and manipulation of the frequency of Tregs

This study was carried out in strict accordance with the recommendations in the Guide for the Care and Use of Laboratory Animals of the National Institutes of Health. The protocol was approved by the Committee on the Ethics of Animal Experiments of the University of Würzburg (Permit Number: 55.2-2531.01-67/06). Specific pathogen free C57BL/6 mice were purchased from Harlan-Winkelmann, Germany. DEREG mice [38] were breeded in the animal facilities of the Institute for Virology and Immunobiology and the Centre for Experimental Medicine, Würzburg. Mice were anesthetized using isofluran and infected intracerebrally (i.c.) into the left hemisphere with 20 µl virus suspension containing 1×10^3^ PFU at an age of two weeks. To expand Tregs, mice were treated intraperitoneally (i.p.) with 100 µg superagonistic anti-CD28 monoclonal antibody (mAb) D665 [36], and to deplete Tregs, DEREG mice were treated i.p. with 1 µg diphtheria toxin (DT; Merck) [38].

### Cell lines and viruses

Vero cells (African green monkey; ATCC CRL 6318) were cultured in Eagle's minimal essential medium (MEM) containing 5% fetal calf serum (FCS), 100 U/mL penicillin and 100 µg/mL streptomycin. Recombinant measles viruses expressing the rodent adapted haemagglutinin of the strain CAM/RB (CAMH) and/or not the enhanced green fluorescent protein (eGFP) rMV^Edtag^EGFP-CAMH (rMV-green) and rMV^Edtag^CAMH (rMV) [35], [45] were propagated using Vero cells.

### Histology

For analyzes, animals were anesthetized with CO_2_ and perfused with 4% (w/v) paraformaldehyde (PFA). Brains were fixed in 4% PFA at least 18 h, and free-floating sections (100 µm) were prepared using a vibratome (Technical Products International) as described [33], [35]. Slices were analyzed directly by UV microscopy. Photomicrographs were taken with a digital camera (Leica). Numbers of infected eGFP-positive neurons were counted and statistical analyzes done using the student's *t* test and the program Prism (GraphPad, Inc.).

### Isolation of lymphocytes from lymph nodes, the spleen, and the brain

Draining cervical lymph nodes and the spleen were pressed through a steel sieve in 4 ml HBSS and homogenized in a total volume of 13 ml HBSS. After a centrifugation step at 310 g for 10 min the cell pellets were resuspended in an adequate volume of HBSS (approximately 10^7^/cells/ml). Spleen cells were additionally treated with erythrocyte lysis buffer (155 mM NH_4_Cl, 10 mM KHCO_3_, 0.1 mM EDTA) and washed with HBSS. Brains were pressed through a steel sieve in 5 ml HBSS 3% FCS and homogenized in a total volume of 20 ml HBSS 3% FCS. After a centrifugation step at 170 g for 10 min the cell pellet was resuspended in 1.4 ml dissociation buffer (23 mM CaCl_2_, 50 mM KCl, 42 mM MgCl_2_, 153 mM NaCl) containing 0.4 U collagenase (Serva) and 50 U Benzonase (Novagen) and incubated at 37°C for 1 h. Afterwards the cells were washed with HBSS and applied on a percoll density gradient to separate the lymphocytes from the rest like myelin debris or neuronal cells as described [46]. The lymphocytes were isolated and washed to remove the percoll for subsequent analyzes.

### Antibodies and flow cytometry

Monoclonal fluorescein-isothiocyanat (FITC)-, phycoerythrin (PE)- or peridinin chlorophyll protein (PerCP)-conjugated anti-mouse CD3 (clone 145-2C11)-, CD4 (clone RM4-5)-, CD8 (clone Ly-2)-, CD19 (clone 1D3)- and CD25 (clone 7D4)-antibodies were purchased from Becton Dickinson. PE-conjugated anti-mouse Foxp3 (clone FJK-16s)-antibody was purchased from NatuTec. Lymphocytes were stained in FACS buffer (PBS containing 0.4% BSA and 0.02% sodium azide) at 4°C for 20 min. Intracellular staining of Foxp3 was performed using the Foxp3 Staining Buffer Set (NatuTec) according to the manufacture's protocol. Briefly, cells were fixed and permeabilized in 500 µl fixation/permeabilization buffer (Concentrate/Diluent 1∶4) at RT for 1 h and stained afterwards in permeabilization buffer at RT for 30 min. Flow cytometric analysis was performed on a FACSCalibur (Becton Dickinson).

### Selection of MHC class I presented peptides

For identification of peptides presented by MHC class I that can be used in ELISPOT and pentamer staining experiments we used the software programs SYFPEITHI (University of Tübingen, Germany) and BIMAS (BioInformatics and Molecular Analysis Section, National Health Instituts, Bethesda, USA) to establish a ranking of potential peptides. From 12 potential H-2 K^b^ and D^b^–presented peptides of MV-N and MV-H with the highest probability scores, we found that D^b^MV-H_22–30_ and D^b^MV-H_446–454_ (RIVINREHL and SNHNNVYWL, respectively) were most efficiently recognized. ELISPOT experiments were performed using the Mouse IFN-γ ELISPOT set (BD Biosciences) according to the manufacture's protocol.

### Pentamer staining

MHC class I (H-2D^b^) pentamers presenting the selected peptide MV-H_22–30_ (D^b^MV-H_22–30_–pentamers) were ordered from ProImmune Ltd (Oxford, UK). Cells were washed with FACS buffer and stained with 5 µL pentamer-solution diluted in 100 µl FACS buffer at 4°C for 30 min. After one washing step the cells were analyzed using the FACSCalibur. MV-specific cells were gated as CD8^+^ and CD19-negative lymphocytes to exclude pentamer^+^ CD19^+^ cells.
